# Toxoplasmosis in Sheep Caused by *Toxoplasma gondii* Clonal Type I

**DOI:** 10.3390/ani15081074

**Published:** 2025-04-08

**Authors:** Yurong Yang, Yiheng Ma, Kai Quan, Bingyan Guo, Yibao Jiang

**Affiliations:** 1 College of Veterinary Medicine, Henan Agricultural University, Zhengzhou 450000, China; yryang@henau.edu.cn (Y.Y.); yihengma@yeah.net (Y.M.); gbingyan123@163.com (B.G.); 2 College of Animal Science, Henan University of Animal Husbandry and Economy, Zhengzhou 450000, China; quankai1115@163.com; 3 College of Animal Science and Technology, Henan Agricultural University, Zhengzhou 450000, China

**Keywords:** ovine abortion, genotypic diversity, epidemiology, ToxoDB # 10, isolation

## Abstract

Ovine toxoplasmosis has a significant negative impact on sheep farming, and abortion is the main clinical manifestation. The objective of the present study was to survey ovine toxoplasmosis and assess its potential harm *T. gondii* in sheep. Sheep serum or heart fluid was collected from 1035 sheep, along with the tissue or blood samples collected from 164 of them. We investigated ovine toxoplasmosis by a modified agglutination test (MAT) (*n* = 1035 sheep) and by a polymerase chain reaction (PCR) (*n* = 164 sheep) in China. The results showed that 75 sheep were seropositive (titer ≥ 1:100), with a prevalence of 7.2%, as assessed by MAT. The molecular prevalence of *T. gondii* in sheep was 16.4% (27/164), as assessed by PCR. From these 164 tissue or blood samples, 22 tissues and 2 blood samples were selected in order to isolate *T. gondii* by mouse bioassay. A viable *T. gondii* strain (TgSheepCHn15, ToxoDB #10) was isolated from sheep tissues in the veterinary clinic. This strain was avirulent for Swiss mice, and the survival time of mice was 97 ± 31 days. This is the first instance of the isolation of a Type I strain from sheep with avirulence in mice. The prevalence of *T. gondii* has been decreasing in sheep from China. However, *T. gondii* remains present in sheep herds and should not be ignored.

## Simple Summary

*Toxoplasma gondii* infects many warm-blooded animals, including sheep. Humans can be infected with *T. gondii* by consuming undercooked sheep tissues containing cysts. Type II (ToxoDB #3 or #1) and Type III (ToxoDB #2) varieties are the epidemic genotypes of viable *T. gondii* strains in sheep. Until now, no viable *T. gondii* Type I (ToxoDB #10) strain has been found in sheep tissues. Type I is usually associated with the highly virulent strain in mice. A viable *T. gondii* strain (TgSheepCHn15), ToxoDB #10, was isolated from the tissues of sheep at a veterinary clinic. This is the first report of a viable *T. gondii* clonal type I genotype that correlates with avirulence in mice.

## 1. Introduction

*Toxoplasma gondii* infects many warm-blooded animals, including sheep. Ovine toxoplasmosis can cause serious reproductive disorders worldwide [1]. Studies have found that ovine toxoplasmosis infection routes include horizontal transmission (ingestion oocysts) and vertical transmission (exogenous or endogenous transplacental transmission) [2,3,4]. Hence, ovine toxoplasmosis can be preserved in sheep herd generations without definitive hosts (felids) [2,4].

In sheep worldwide, Type II (ToxoDB #3 or #1, 63%) and Type III (ToxoDB #2, 9%) are the epidemic genotypes of viable *T. gondii* strains [5]. Until now, no viable *T. gondii* Type I (ToxoDB #10) strain has been found in sheep tissues. Usually, Type I is associated with a highly virulent mouse strain, such as the RH strain or GT1 strain [6,7]. In this study, the *T. gondii* clonal type I genotype that correlates with avirulence in mice was found in sheep. China currently ranks the highest in sheep and goat meat production (animal production: 5166 kilotons in 2022). However, there has been limited research on ovine toxoplasmosis. The objective of the present study was to survey ovine toxoplasmosis and assess its potential harm to the host.

## 2. Materials and Methods

### 2.1. Information on Samples and Sources

**Figure 1:**
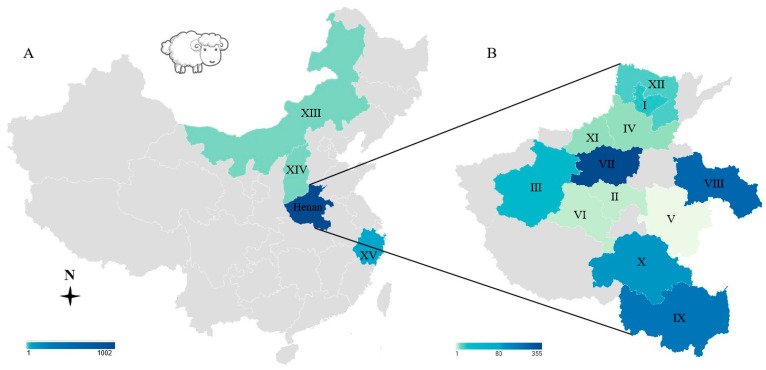
Locations in China of the samples received. (**A**): China; (**B**): Henan. I: Hebi, II: Xuchang, III: Luoyang, IV: Xinxiang, V: Zhoukou, VI: Pingdingshan, VII: Zhengzhou, VIII: Shangqiu, IX: Xinyang, X: Zhumadian, XI: Jiaozuo, XII: Anyang, XIII: Inner Mongolia, XIV: Shanxi, XV: Zhejiang.

From 2018 to 2024, samples from 1035 sheep were obtained from Henan, Shanxi, Inner Mongolia, and Zhejiang provinces (Figure 1, Table 1). Samples included tissues (84 myocardial tissues from the slaughterhouses; the heart, liver, spleen, lungs, kidney, and lymphonodus of 12 lambs from the slaughterhouses; the heart, liver, spleen, lungs, kidney, and lymphonodus of 38 dead sheep or aborted fetuses from veterinary clinics) from 134 sheep, as well as heart juice (*n* = 134), sera (528 sera from the farms, 323 sera from the slaughterhouses, 20 sera from veterinary clinics, *n* = 871) and blood (*n* = 30, from veterinary clinics) from 1035 sheep. The samples were sent to the laboratory (Veterinary Pathology, Henan Agricultural University, Zhengzhou, Henan Province, China) for etiological diagnosis, the evaluation of meat quality, and to check the *Brucella* spp. infection status. The sheep were divided into three groups according to the sample source (farms, veterinary clinics, and slaughterhouses). For the supervision of *Brucella* spp. infection status, we only obtained serum samples from alive sheep in farms, with ages ranging from 1 month to 6 years old; these samples were defined as having a “farm source”. For meat quality evaluation, tissues or serum samples were obtained from slaughterhouses using animals ranging in age from 8 to 10 months and showing good clinical health. For etiological diagnosis, tissues or serum samples were collected from sick sheep, or aborted fetuses, or alive sheep with the clinical suspicion of disease, with the age of animals ranging from 0 days to 6 years old; these samples were defined as having a “veterinary clinics source”. The survey of ovine toxoplasmosis in these sheep was also allowed. These samples were not collected according to the epidemiological survey software; they were scattered.

### 2.2. Detection of T. gondii Antibodies in Sheep

For *T. gondii*, the postmortem detection of antibodies in meat juice samples is a useful alternative to serum examination [8,9]. In this study, for samples from slaughterhouses or dead cases, blood was removed from each heart and centrifuged to obtain the supernatant, which was termed “heart juice”. Heart juice (*n* = 134) and serum samples (*n* = 901) were tested for *T. gondii* IgG antibodies by a modified agglutination test (MAT) [10,11,12]. Titers ≥ 1:100 indicated *T. gondii* infection, as compared to viable parasite isolation from sheep tissues [11,12,13]. Formalin-fixed *T. gondii* RH tachyzoites were provided by the University of Tennessee (Knoxville, TN, USA). *T. gondii-*infected mice serum was used as a reference.

### 2.3. T. gondii Nucleic Acid Detection in Sheep

DNA was extracted from blood (30 sheep) or tissue samples (134 sheep) by a DNA extraction kit (Tiangen Biotec Company, Beijing, China, DP304). *T. gondii* was examined using the primer pair TOX-5 (5′-CGC TGC AGA CAC AGT GCA TCT GGA TT-3′) and TOX-8 (5′-CCC AGC TGC GTC TGT CGG GAT-3′) [14]. Target DNA was amplified using the following program: initial denaturation at 94 °C for 5 min, denaturation at 94 °C for 1 min, annealing at 60 °C for 1 min, and extension at 72 °C for 1 min. Following 35 cycles, a final extension step of 10 min was added at 72 °C. The PCR product was 450 bp, and negative (negative samples were from *Toxoplasma gondii* nucleic acid detection kit, Product ID: 25T, Shanghai Yan Qi Biological Technology Company, Shanghai, China) and positive (VEG strain infected mice brain was provided by Dr. Dubey) [15] controls were included in every batch.

### 2.4. Morphological Observation of T. gondii Parasites in Sheep Tissues

Tissue samples (only myocardial tissues from 84 sheep; heart, liver, spleen, lungs, kidney, and lymphonodus of 12 lambs; heart, liver, spleen, lungs, kidney, and lymphonodus of 38 dead sheep or aborted fetuses) from 134 sheep were fixed using formalin, embedded in paraffin, stained with hematoxylin and eosin, and subjected to immunohistochemistry (IHC) for *T. gondii* [1,16]. *T. gondii*-infected mouse tissues and *T. gondii*-free mouse tissues were used as positive and negative controls, respectively.

### 2.5. Isolation Viable T. gondii from Tissues of Sheep

Mouse bioassays were performed on blood or tissue samples of sheep (Table 1) . From 134 and 30 blood samples, 22 tissues and 2 blood samples were selected to isolate *T. gondii* by a mouse bioassay. Via a mouse bioassay, we used serologies or etiologies positive for *T. gondii* as selection references for most samples. Some samples with serologies or etiologies negative for *T. gondii* were also selected to isolate viable strains.

In brief, tissue (striated muscles or tissues containing *T. gondii* DNA, 50 g) was ground or pepsin-digested and used to inoculate Swiss mice (*n* = 2–5) or a C57BL/6 IFN-γ^−/−^ mouse subcutaneously [1]. Clinical signs were observed. *T. gondii* parasites were examined in the lungs, mesenteric lymph nodes, pleural effusion, ascites, or the brain of dead mice. Facial vein blood was obtained from mice 30 days post-inoculation (DPI) and 60 DPI, and 1:100 dilutions of serum were checked for *T. gondii* antibodies by MAT. If no antibodies and parasites were found, the homogenized mouse lungs, brain, spleen, and heart were subpassaged into a new mouse group subcutaneously. Only one round of mice was inoculated with negative samples.

### 2.6. Cell Cultivation and Genotyping

The mouse lungs were seeded into Vero cells (ThermoFisher, Waltham, MA, USA). DNA was extracted from *T. gondii*-infected mice tissues. Then, PCR-RFLP was used to check the genotype of the *T. gondii* isolate using 10 genetic markers [17]. The virulent genes ROP18 and ROP5 were also typified [18]. Eight *T. gondii* DNA (obtained from Dr. Chunlei Su, University of Tennessee) samples were used for reference.

### 2.7. Statistical Analysis

Data are expressed as the mean ± SEM using GraphPad Prism 8.0, followed by one-way ANOVA, with *p* < 0.05 considered to be statistically significant. Statistical analyses relating to organelles in the isolated *T. gondii* strain were performed using transmission electron microscopy (TEM). *T. gondii* genotype analysis was performed by SplitsTree CE 6.0.0 [19]. The original input consisted of 56 taxa and 57 standard sequences of length 62. The Show Splits method (default options) was used to obtain Split Network visualization. To explore the phylogenetic position of the strain studied, strains (*n* = 48) from Central China were selected, with the background of the strains shown in Table S1.

## 3. Results

### 3.1. Investigation of Ovine Toxoplasmosis in Sheep

In this survey, we checked heart juice (*n* = 134) or sera (*n* = 901) from 1035 sheep for *T. gondii* IgG antibodies by MAT. Overall, 7.2% (75/1035) (95% confidence interval 5.81–9.00%) of sheep had *T. gondii* antibodies (Table 1 and Figure 1). The titers of these samples ranged from 1:100 to 1:12,800, with a titer of 1:100 in 12 sheep, 1:200 in 20, 1:400 in 2, 1:800 in 7, 1:1600 in 15, 1:3200 in 11, 1:6400 in 5, and 1:12800 in 3.

The sheep were divided into three groups according to the sample source. Namely, farms, veterinary clinics, and slaughterhouses. The *T. gondii* prevalence in sheep was 9.8% (52/528, 95% CI: 7.57–12.70%) in farms, 7.1% (12/169, 95% CI: 4.00–12.11%) in veterinary clinics, and 3.3% (11/338, 95% CI: 1.76–5.80%) in slaughterhouses. Compared with sheep in slaughterhouses, the prevalence of *T. gondii* was higher in farms or veterinary clinics (*p <* 0.05), with an odds ratio of 3.248 or 2.272 (Table 1 and Table 2).

The prevalence of *T. gondii* in sheep was higher in the <1-year group (16.1%, 61/378) compared to the >1-year group (2.1%, 14/657) (*p* < 0.0001). When analyzed by geographic location, there were few samples from Inner Mongolia (*n* = 1), Shanxi (*n* = 1), and Zhejiang (*n* = 31), and none of them showed seroprevalence for *T. gondii* (Table 1 and Table 2).

According to the PCR results, *T. gondii* DNA was detected in 27 of 164 sheep (16.4%, 95% CI: 11.52–22.94%). These included whole blood (*n* = 11), multiple organs of two ewes, three lambs, three aborted fetuses, and one placenta from veterinary clinics; myocardium samples (*n* = 7) from slaughterhouses; and none of the sheep from farms (Table 1).

### 3.2. Assessment of Pathological Lesions, and T. gondii Antigen Distribution

**Figure 2:**
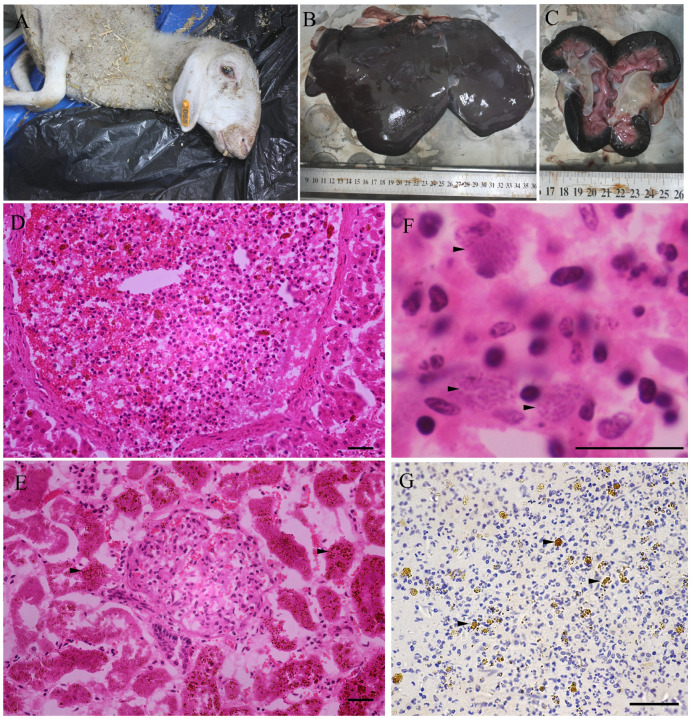
Pathological findings from sheep. (**A**) On 11 October 2021, a ewe from the veterinary clinic showed emaciation and anemia. (**B**) The liver was swollen, crisp, and dark purple, and the gallbladder was enlarged and full of bile. (**C**) The kidneys were atrophic and black, and the fat was jelly-like. (**D**) The hepatic veins were filled with neutrophils, HE. (**E**) Necrotizing atrophic renal tubular nephritis, huge hemoglobin deposits in renal tubular epithelial cells (arrowhead), HE. (**F**) Necrotizing lymphadenitis, with many macrophages in lymph nodes with *Toxoplasma gondii*-like parasites (arrowhead), HE. (**G**) Anti-*T. gondii*-antibody staining in lymph nodes tissue (arrowhead), IHC. Bar: 50 μm.

Tissue samples were obtained from 134 sheep between 2018 and 2024 (Table 1). TgSheepCHn15 was isolated from the tissue of a ewe (Xinxiang, Henan, 11 October 2021) (Figure 2). The ewe had a moderate appetite, emaciation, and anemia, could ruminate, and died the next day. Necropsy showed that the lungs had adhered to the wall of the chest, with pulmonary congestion and white foam observed in the trachea. The liver was swollen, crisp, and dark purple in color, and the gallbladder was enlarged and full of bile. The spleen was mildly enlarged, soft, and black in color. The kidneys were atrophic and black, and the fat was yellow and jelly-like. The mesenteric lymph nodes were enlarged with striated black spots on the cut surface. Microscopically, the interlobular boundaries of the liver were unclear, and the hepatic cords were arranged in a disordered manner. The hepatic veins were filled with neutrophils. Necrotizing lymphadenitis was observed, with many macrophages in the lymph nodes with *T. gondii*-like parasites. *T. gondii* parasites were verified in this ewe by immunohistochemistry (Figure 2). Interstitial pneumonia and necrotizing splenitis were observed. Necrotizing atrophic renal tubular nephritis and huge hemoglobin deposits were observed in renal tubular epithelial cells. Pathological changes suggested that this ewe had chronic hemolytic anemia (copper poisoning, proven by mineral element testing), with a history of exposure to *T. gondii*.

*T. gondii* parasites were also observed in the lungs of a lamb from Zhumadian, Henan (13 September 2021) by IHC, while *T. gondii* DNA was also detected in the lungs and diaphragm of this ewe. *T. gondii* parasites were not observed in paraffin sections of 132 other sheep.

### 3.3. Isolation of the T. gondii Strain from Sheep Tissues or Blood

**Figure 3:**
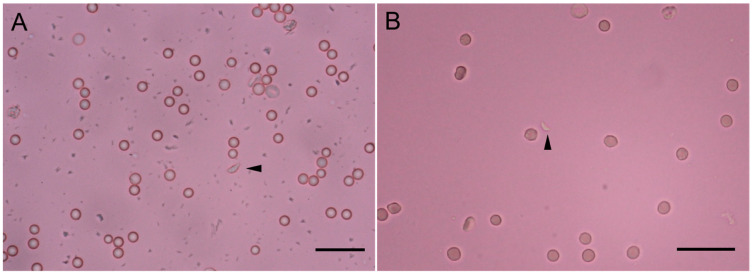
The morphology of the TgSheepCHn15 strain. (**A**) A tachyzoite (arrowhead) was found in the lung of TOX#35-43#629 Swiss mouse, 15 days post-inoculation (DPI), unstained smear. (**B**) A tachyzoite (arrowhead) was found in the lung of TOX#35-46#643 Swiss mouse, 19 DPI, smear, unstained. Bar: 20 μm.

We selected tissue samples and the blood of 24 sheep to isolate *T. gondii* by mouse bioassay (Table 1). In the Tox#35-43 group (“Tox#35” indicates samples from sheep, “43” is the group number), three Swiss mice (M#628, M#629, and M#630) were inoculated with the digestive fluid of the skeletal muscles, liver, and spleen from the ewe (sampling from 11 October 2021, Xinxiang) (Figure S1). These three mice died at 15–27 DPI with clinical manifestations of depression and poor appetite, and tachyzoites were found in the lungs of the M #629 mice (Figure 3A). In the Tox#35-44 group, five Swiss mice (M#631-M#635) were inoculated with ground tissues from the lymph nodes and spleen of the ewe (sampling from 11 October 2021, Xinxiang). These mice died of bacteremia 2–4 DPI. Tox#35-45, Tox#35-46, and Tox#35-47 were inoculated with the myocardium, lungs, spleen, and lymph nodes of Tox#35-44 mice. *T. gondii* parasites were observed in the lungs of M #643 (Tox#35-46) at 19 DPI (Figure 3B). Tox#35-48 and Tox#35-49 were inoculated with the tissues of Tox#35-45 and Tox#35-47 mice, respectively. The 50% mice were seropositive for *T. gondii* (≥1:200) when using MAT in the Tox35-48 (M #654) and Tox35-49 (M #659) groups at 30 DPI. Tachyzoites were observed in the lymph nodes and lungs of M#654 at 32 DPI. The survival time for M#659 was 116 DPI, and no brain cysts were observed.

The brain, lymph nodes, and lungs of M#654 mice were homogenized and inoculated into the mice groups and seeded into Vero cells. This *T. gondii* strain could propagate slowly in cell culture at 16 DPI, and bradyzoites could be formed in the cell culture medium at 50 DPI. In vivo, only 42.9% of the inoculated mice groups (9/21) could infect *T. gondii* (25–50% mice infected with *T. gondii* in positive groups, verified by MAT or crescent-shaped parasites). This strain was designated as TgSheepCHn15.

In the Tox#35-41 group (lamb samples from 23 September 2021, Zhoukou), 1/5 mice (M#789) were seropositive for *T. gondii*, and parasites were observed in the IFN-γ^−/−^ mice M#727 lungs at 6 DPI. Unfortunately, none of the mice were exposed to *T. gondii* after being sub-passaged.

No viable *T. gondii* strain was isolated from other sheep (*n* = 22).

### 3.4. Genotypes of TgSheepCHn15

The TgSheepCHn15 genotype was determined from the heart, lungs, and lymph nodes of M#654 (Tox#35-48), and it belonged to ToxoDB genotype #10 (Figure S2, Table S2). The ROP18/ROP5 allele type was 1/1 (Figure S2). Infection with the TgSheepCHn15 strain was in all of the inoculated mice. According to the survival time (97 ± 31) of infected mice (Table 3), the TgSheepCHn15 strain was avirulent to Swiss mice.

**Figure 4:**
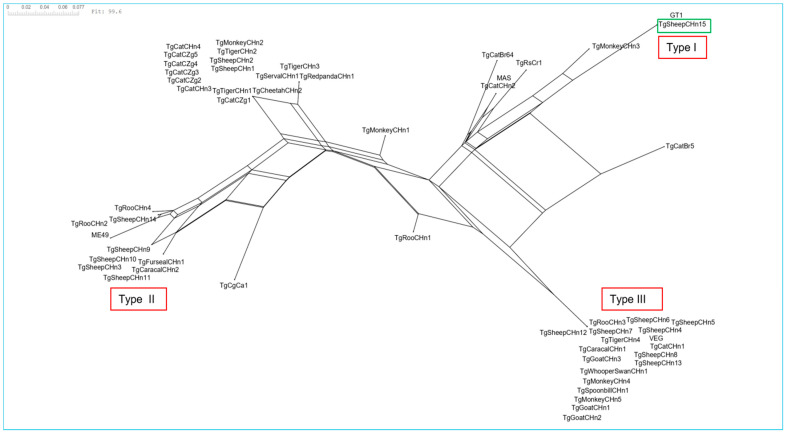
Neighbor-joining clustering of *Toxoplasma gondii* by 10 PCR-RFLP markers. The green box is the TgSheepCHn15 strain. Other *Toxoplasma gondii* strains from central China were chosen (Table S1). GT1, ME49, VEG, TgCgCal, MAS, TgCatBr5, TgCatBr64, and TgRsCr1 are reference strains.

The genotyping of IFN-γ^−/−^ mouse M #727 (Tox35-41, lamb samples from 23 September 2021, Zhoukou) tissues using PCR-RFLP was unsuccessful. The neighbor-joining analysis based on PCR-RFLP data indicated that TgSheepCHn15 is disparate from other reported Chinese strains genotyped with PCR-RFLP 10 markers (Figure 4).

## 4. Discussion

A viable *T. gondii* TgSheepCHn15 strain was isolated from tissues of a ewe (heart juice titer = 1:25) in a veterinary clinic (Table 1). The method of grinding tissues (lymph nodes and spleen) was superior to pepsin digestion (skeletal muscles, liver, and spleen). *T. gondii* bradyzoites were observed in the mesenteric lymph node of the ewe (Figure 2), indicating that it might be in the acute stage of infection, supported by molecular biology evidence (*T. gondii* DNA was detected in the liver, lungs, and blood) and morphological evidence obtained by IHC.

The *T. gondii* TgSheepCHn15 genotype was ToxoDB #10 (Type I). In China, some researchers have reported the isolation and genotypes of *T. gondii* in sheep. Between 2019 and 2021, the *T. gondii* strain (ToxoDB #3) was isolated from sheep hearts [16]. From 2017 to 2019, 11 *T. gondii* isolates were obtained from sheep hearts. Seven isolates were ToxoDB#2 and four isolates were ToxoDB #4 [12]. From 2014 to 2016, two *T. gondii* strains were obtained from sheep hearts, both of which were ToxoDB #9 [20]. The genotype of the *T. gondii* strain in sheep from Qinghai was ToxoDB #3 [21]. *T. gondii* TgSheepCHn15 could grow and be sub-passaged in Vero cells and mice, but showed poor growth performance, possibly due to the characteristics of this strain. However, the exploration of the organelle structure by TEM was unsuccessful for a smaller number of *T. gondii*.

*Toxoplasma gondii* ToxoDB #10 strains and DNA seen worldwide are summarized in Table S3. Viable *T. gondii* ToxoDB #10 strains with high pathogenicity have been isolated from humans, goats, cats, and chickens in the USA, Brazil, Colombia, and China [1]. ToxoDB #10 nucleic acids have been found in aborted sheep fetuses from Iran [22]. Furthermore, ToxoDB #10 nucleic acids have been isolated from pigs, *Microtus fortis*, goats, cattle, donkeys, chickens, sparrows, foxes, raccoon dogs, and bats from China [1]. These findings were not based on viable parasites, and their virulence is unknown. However, some samples were from slaughterhouses or animals that appeared to be clinically healthy, indicating that they were avirulent for the hosts.

More than 80% of the 1/1 ROP18 and ROP5 alleles in *T. gondii* strains are lethal [18]. The ROP18 and ROP5 alleles of TgSheepCHn15 (1/1) were not lethal in mice (the survival time of TgSheepCHn15 infected mice was 97 ± 31 days), which is consistent with the same type of TgCtCo2 from Colombia [23]. Furthermore, no *T. gondii* TgSheepCHn15 cysts were found in the brain of mice (as identified by MAT and PCR). This may be related to the characteristics of the host and the inoculated parasites [24]. The genetic evolutionary distance of TgSheepCHn15 from other *T. gondii* strains isolated in Central China is significant, as shown in Figure 4. Overall, TgSheepCHn15 is distinctly different from the other ToxoDB #10 strains.

Ovine toxoplasmosis can cause serious economic losses worldwide [1,5,13]. Recently, the prevalence of *T. gondii* has been decreasing in sheep from China. However, *T. gondii* remains present in sheep herds and should not be ignored. The *T. gondii* prevalence in sheep from Henan Province from 2018 to 2024 was 7.5%, which was lower than that seen from 2017 to 2019 (25.3%) [12]. Both studies used 1:100 as a cut-off value of MAT. The low prevalence of *T. gondii* in sheep may be related with the development of the intensive management and health management of the sheep industry in China [25]. The seroprevalence of *T. gondii* in <1-year-old sheep (16.1%) was higher than that in older sheep (2.1%). This phenomenon may be related to the sample source (young sheep were mainly from veterinary clinics, and old sheep were mainly from slaughterhouses or farms) or the limited number of samples. Furthermore, the prevalence of ovine toxoplasmosis in farms or veterinary clinics was higher than that in slaughterhouses. Some unhealthy sheep enter veterinary clinics or pathology laboratories because of toxoplasmosis or other pathogens. Sheep infected with other pathogens or exhibiting sickness may also have an increased chance of infection with *T. gondii.* Generally, the *T. gondii* prevalence in sheep increased with age, indicating that ingestion oocysts constitute the main transmission route for sheep [3,5].

For chronic toxoplasmosis, the rate of isolation *T. gondii* or the number of tissue cysts was correlated with antibody titers in sheep and mice [11,12,26]. In this study, *T. gondii* DNA was found in 27 tissues or the blood of 164 sheep; only one sheep showed *T. gondii* antibodies (MAT titer = 1:200) from slaughterhouses, and the other 26 sheep were all <1:25 (6 sheep were from slaughterhouses, and 20 sheep were from veterinary clinics). MAT can detect IgG acting against *T. gondii* at the stage of chronic infection [10], indicating the need for additional examination for IgM antibodies or the etiological assessment of samples from veterinary clinics or sheep that may be in the acute stage of infection. *T. gondii* can be isolated from seronegative or etiological negative (low density of *T. gondii* parasites) animal tissues [27,28]. The findings of this study indicate that the parasite load density has no correlation with antibody titers in sheep at the stage of acute toxoplasmosis.

## 5. Conclusions

In the present study, one *T. gondii* clonal type I (ToxoDB #10) strain was isolated from adult sheep tissues. To the best of our knowledge, this is the first viable *T. gondii* ToxoDB #10 strain reported in sheep worldwide, and it is not lethal to mice. Further research is needed for the impact of TgSheepCHn15 and other isolated strains on sheep health and potential zoonotic risks.

## Figures and Tables

**Table 1:** Isolation and prevalence of *Toxoplasma gondii* from sheep (2018–2024).

Provinces	Location ^a^	Source	Sample Received Date	No. of Samples	Positive /Test by MAT ^b^	Positive/Test by PCR ^c^, Positive Tissues	Positive /Test by IHC ^d^	Isolation by Mice ^e^	Mice Group Tox# ^f^
Inner Mongolia	XIII	Vet	12 Dec 2018	1 lamb	0/1	0/1	0/1		
Shanxi	XIV	Vet	14 Jan 2019	1 lamb	0/1	0/1	0/1		
Henan *n* = 1002	I: Hebi	Vet	6 May 2019	8 ewe sera, 11 aborted feuses	0/19	0/11	0/11		
	II: Xuchang	Sh	15 May 2019	2 lambs	2/2	0/2	0/2	0/2	5-306, 307
	III: Luoyang	Vet	14 Sep 2021	2 lambs	0/1	0/2	0/2	0/1	35-40
		Vet	15 Sep 2021	2 lambs	2/2	0/2	0/2		
		Sh	15 Jun 2023	35 lamb hearts	1/35	2/35	0/35	0/3	35-81-35-83
	IV: Xinxiang	Vet	11 Oct 2021	1 ewe	0/1	1/1, Li, Lu, Bl	1/1, LN	1/1 TgSheepCHn15	35-43-35-50, 35-53, 55, 56, 58
			3 Dec 2021	2 aborted fetus, placenta	0/2	2/2, Pl, H, Li, Lu, Um	0/2		
			19 Dec 2021	3 aborted fetus, 1 placenta	0/3	0/3	0/3		
			14 Mar 2022	1 aborted fetus, 1 placenta	0/1	1/1, Pl	0/1		
			25 Mar 2024	1 ewe	0/1	0/1	0/1		
	V: Zhoukou	Vet	23 Sep 2021	1 lamb	1/1	0/1	0/1	1/1	35-41, 42
	VI: Pingdingshan	Vet	12 Nov 2021	2 lambs	0/2	1/2, H, Sk	0/2		
	VII: Zhengzhou	Vet	9 Dec 2021	1 lamb	0/1	0/1	0/1		
		Sh	30 May 2023	41 lamb hearts	3/41	5/41	0/41	0/8, 3 hearts pool	35-71-35-78
		Farm	2 May 2021	313 ewe sera	0/313	nd	nd		
	VIII: Shangqiu	Vet	22 Mar 2022	9 ewe sera, 1 ram serum	0/10	nd	nd		
		Sh	2 Apr 2023	242 ewe sera	5/242	nd	nd		
		Vet	26 May 2022	1 lamb	0/1	1/1, Bi, Bla	0/1		
		Vet	16 Jan 2024	1 lamb	0/1	0/1	0/1		
	IX: Xinyang	Farm	16 Mar 2022	45 lamb sera	12/45	nd	nd		
			29 Mar 2022	15 lamb sera	1/15	nd	nd		
			22 Apr 2022	29 lamb sera	0/29	nd	nd		
			18 May 2022	30 lamb sera	2/30	nd	nd		
			18 Jun 2022	30 lamb sera	29/30	nd	nd		
			29 Sep 2022	31 lamb sera	8/31	nd	nd		
			11 Oct 2022	35 lamb sera	0/35	nd	nd		
	X: Zhumadian	Vet	13 Sep 2021	1 lamb, 2 sera	0/3	1/1, Lu, Di	1/1, Lu		
			26 Sep 2021	31 ewe sera	3/31	nd	nd		
			16 Dec 2021	50 ewe sera	6/50	nd	nd		
			16 Nov 2023	2 lambs	0/2	0/2	0/2		
	XI: Jiaozuo	Sh	21 Nov 2023	8 lamb hearts	0/8	0/8	0/8	0/6	35-84-35-89
	XII: Anyang	Vet	23 Jan 2024	1 ewe, 2 aborted fetuses	0/3	2/3, H, Li, Lu, Ki, Sk, T, Br/ewe; Li, Lu, Ki, Sk, T, Br 1/2 fetuses	0/3		
		Sh	30 Apr 2024	10 lambs	0/10	0/10	0/10		
Zhejiang	XV	Vet	4 Aug 2022	30 lamb blood	0/30	11/30	nd	0/2	35-64,65
			10 Dec 2023	1 lamb	0/1	0/1	0/1		
Overall prevlence			Tissues from 134 sheep Sera or blood from 901 sheep		7.2% (75/1035)	16.4% (27/164)	1.5% (2/134)	2/24	

^a^: Figure 1 shows sampling cities; ^b^: MAT: modified agglutination test; ^c^: PCR: polymerase chain reaction; ^d^: IHC: immunohistochemistry; ^e^: number of positive isolates/number of sheep; ^f^: *T. gondii* isolation group number. nd: not done; Vet: veterinary clinic; Sh: slaughterhouse; Bi: bile; Bl: blood; Bla: bladder; Br: brain; Di: diaphragm; H: heart; Ki: kidney; Li: liver; Lm: Leg muscle; LN: lymph node; Lu: lung; Pd: pepsin digest juice of striated muscles; Pl: placenta; Sp: spleen; Sk: skeletal muscle; T: tongue; Um: umbilical cord.

**Table 2:** Prevalence and risk factors for *Toxoplasma gondii* in sheep as determined using modified agglutination test.

	Variable	Number of Samples	Seropositivity (n/%)	Odds ratio (95% Confidence Internal)	*p* Value
Sampling sites	Farm	528	52/9.8	3.248 (1.669–6.318)	0.0003 *
	Veterinary clinic	169	12/7.1	2.272 (0.9809–5.263)	0.0498 *
	Slaughterhouse	338	11/3.3	1	
Age (y)	<1	378	61/16.1	8.838 (4.869–16.04)	<0.0001 *
	≥1	657	14/2.1	1	
Location	Henan	1002	75/7.5	-	-
	Inner Mongolia	1	0/1	-	-
	Shanxi	1	0/1	-	-
	Zhejiang	31	0/31	-	-

* statistically significant.

**Table 3:** Survival time of Swiss mice infected withTgSheepCHn15.

Group# ^a^	Mouse# ^b^	Serum MAT Titer/DPI	Survival Time (Days)
35-43	629	Tachyzoites were observed in lungs/15	15
35-46	643	Tachyzoites were observed in lungs/19	19
35-45	639	≥1:100 (liver juice)/13	13
35-48	654	≥1:200/30	32
35-49	659	≥1:200/30	≥116
35-55	689	≥1:200/30	≥102
35-56	912	≥1:200/30	≥81
35-58	947	≥1:200/52	214
35-58	948	≥1:200/52	≥278
Mean ± SEM			97 ± 31

^a^: *Toxoplasma gondii* isolation group number; ^b^: mouse number.

## Data Availability

The datasets used and/or analyzed are available from the corresponding author upon reasonable request.

## Supplementary Materials

The following supporting information can be downloaded at https://www.mdpi.com/article/10.3390/ani15081074/s1. Figure S1: Flow chart for isolation of *Toxoplasma gondii* from sheep tissues; Figure S2: Genotypes of *Toxoplasma gondii* strain from sheep; Table S1: SplitsTree data of viable *T. gondii* isolates (*n* = 48) from animals in Central China. Table S2: Genotypes of *Toxoplasma gondii* isolate from sheep by PCR-RFLP; Table S3: Isolation *Toxoplasma gondii* ToxoDB #10 (strains or DNA) from human and animals (1937–2024).

## Abbreviations

The following abbreviations are used in this manuscript:

DPI	days post-inoculation
MAT	modified agglutination test
PCR	polymerase chain reaction
IHC	immunohistochemistry
