# Supplementary material for: Toxoplasmosis in Sheep Caused by Toxoplasma gondii Clonal Type I
Source: Animals (Basel). 2025 Apr 8;15(8):1074. doi: 10.3390/ani15081074 (PMC12024269; doi:10.3390/ani15081074)
Supplement: Supplementary file 1 [file animals-15-01074-s001.zip › animals-3499632-supplementary.pdf]

Table S1. SplitsTree data of viable *T. gondii* isolates (n=48) from animals in Central China.

**Species**	**Number**	**Strain ID**	**Genotype ToxoDB#**	**References**
Felinae	8	TgCatCHn1-3, TgCatCZg1-5	6 #9, 1 #2, 1 #17	1
Felinae	1	TgCatCHn4	#9, 1	2
Ovis aries	2	TgSheepHn1-2	All #9	3
Ovis aries	11	TgSheepHn3-13	7 #2, 4 #4	4
Ovis aries	1	TgSheepHn14	#3	5
Ovis aries	1	TgSheepHn15	#10	This study
Macropus giganteus	1	TgRooCHn1	#292	6
Macropus rufogriseus	1	TgRooCHn2	#3	7
Macropus rufus	1	TgRooCHn3	#2	7
Macropus rufogriseus	1	TgRooCHn4	#3	8
Ailurus fulgens	1	TgRedpandaCHn1	#20	9
Leptailurus serval	1	TgServalCHn1	#20	10
Panthera tigris	2	TgTigerCHn1-2	All #9	11
Panthera tigris	2	TgTigerCHn3-4	#20, #2	12
Caracal caracal	1	TgCaracalCHn1	#2	13
Caracal caracal	1	TgCaracalCHn2	#5	14
Platalea leucorodia	1	TgSpoonbillCHn1	#2	15
Macaca mulatta	1	TgMonkeyCHn1	Mix	16
Erythrocebus patas	1	TgMonkeyCHn2	#9	17
Lophocebus aterrimus	1	TgMonkeyCHn3	#6	Ma et al., unpublished
Erythrocebus patas	2	TgMonkeyCHn4-5	#2	unpublished
Acinonyx jubatus	1	TgCheetahCHn2	#9	18
Arctocephalinae	1	TgFursealCHn1	#5	Mao et al., unpublished
Capra hircus	3	TgGoatCHn1-3	#2	unpublished
Cygnus cygnus	1	TgWhooperSwanCHn1	#2	unpublished
	N=48			

Table S1 References

1.Yang, Y.; Ying, Y.; Verma, S.K; Cassinelli, A.B.; Kwok, O.C.; Liang, H.; Pradhan, A.K.; Zhu, X.; Su, C.; Dubey, J.P. Isolation and genetic characterization of viable *Toxoplasma gondii* from tissues and feces of cats from the central region of China. *Vet. Parasitol.* **2015**, 211(3-4), 283-288.

2. Yang, Y.; Feng, Y.; Lu, Y.; Dong, H.; Li, T.; Jiang, Y.; Zhu, X.; Zhang, L. Antibody detection, isolation, genotyping, and virulence of *Toxoplasma gondii* in captive felids from China. *Front. Microbiol.* **2017**, 8,1414.

3. Yang, Y.; Feng, Y.; Yao, Q.; Wang, Y.; Lu, Y.; Liang, H.; Zhu, X.; Zhang, L. Seroprevalence, isolation, genotyping, and pathogenicity of *Toxoplasma gondii* strains from sheep in China. *Front. Microbiol.* **2017**, 8: 136.

4. Jiang, N.; Su, R.; Jian, F.; Su, C.; Zhang, L.; Jiang, Y.; Yang, Y. *Toxoplasma gondii* in lambs of China: heart juice serology, isolation and genotyping. *Int. J. Food Microbiol*. **2020**, 322: 108563.

5. Jiang, Y.; Xin, S.; Ma, Y.; Zhang, H.; Yang, X.; Yang, Y. Low prevalence of *Toxoplasma gondii* in sheep and isolation of a viable strain from edible mutton from Central China. *Pathogens.* **2023**, 12(6): 827.

6. Su, R.; Dong, H.; Li, T.; Jiang, Y.; Yuan, Z.; Su, C.; Zhang, L.; Yang, Y. *Toxoplasma gondii* in four captive kangaroos (*Macropus* spp.) in China: isolation of a strain of a new genotype from an eastern grey kangaroo (*Macropus giganteus*). *Int. J. Parasitol. Parasites Wildl.* **2019**, 8, 234-239.

7. Yang, L.; Xin, S.; Zhu, N.; Li, J.; Su, C.; Yang, Y. Two viable *Toxoplasma gondii* isolates from red-necked wallaby (*Macropus rufogriseus*) and red kangaroo (*M. rufus*). *Parasitol. Int.* **2023**, 92, 102687.

8. Yang, L.; Ren, H.; Zhu, N.; Mao, G.; Li, J.; Su, C.; Jiang, Y.; Yang, Y. Epidemiology and isolation of viable *Toxoplasma gondii* strain from macropods. *Heliyon.* **2023**, 9(3), e13960.

9. Yang, Y.; Dong, H.; Su, R.; Li, T.; Jiang, N.; Su, C.; Zhang, L. Evidence of red panda as an intermediate host of *Toxoplasma gondii* and *Sarcocystis* species. *Int. J. Parasitol. Parasites Wildl.* **2019**, 8, 188-191.

10. Dong, H.; Su, R.; Li, T.; Su, C.; Zhang, L.; Yang, Y. Isolation, genotyping and pathogenicity of a *Toxoplasma gondii* strain isolated from a Serval (*Leptailurus serval*) in China. *Transbound Emerg. Dis.* **2019**, 66(4), 1796-1802.

11. Yang, Y.; Dong, H.; Su, R.; Jiang, N.; Li, T.; Su, C.; Yuan, Z.; Zhang, L. Direct evidence of an extra-intestinal cycle of *Toxoplasma gondii* in tigers (*Panthera tigris*) by isolation of viable strains. *Emerg. Microbes Infect.* **2019**, 8(1), 1550-1552.

12. Ren, H.; Yang, L.; Zhu, N.; Li, J.; Su, C.; Jiang, Y.; Yang, Y. Additional evidence of tigers (*Panthera tigris altaica*) as intermediate hosts for *Toxoplasma gondii* through the isolation of viable strains. *Int. J. Parasitol. Parasites Wildl.* **2022**, 19, 330-335.
13. Jiang, N.; Xin, S.; Li, J.; Su, C.; Zhang, L.; Yang, Y. Isolation and characterization of *Toxoplasma gondii* from captive caracals (*Caracal caracal*). *Int. J. Parasitol. Parasites Wildl.* **2020**, 13, 196-201.
14. Ren, H.; Mao, G.; Zhang, Y.; Zhu, N.; Liang, Q.; Jiang, Y.; Yang, Y. Isolation and characterization of a viable *Toxoplasma gondii* from captive caracal (*Caracal caracal*). *Pathogens.* **2023**, 12(12), 1412.
15. Yang, Y.; Jiang, N.; Xin, S.; Zhang, L. *Toxoplasma gondii* infection in white spoonbills (*Platalea leucorodia*) from Henan Province, China. *Emerg. Microbes Infect.* **2020**, 9(1), 2619-2621.
16. Xin, S.; Jiang, N.; Yang, L.; Zhu, N.; Huang, W.; Li, J.; Zhang, L.; Su, C.; Yang, Y. Isolation, genotyping and virulence determination of a *Toxoplasma gondii* strain from non-human primate from China. *Transbound Emerg. Dis.* **2022**, 69(2), 919-925.
17. Yang, L.; Ren, H.; Zhu, N.; Xin, S.; Mao, G.; Ma, Y.; Li, J.; Liang, Q.; Yang, Y. Isolation and genetic characterization of *Toxoplasma gondii* from a Patas Monkey (*Erythrocebus patas*) in China. *Genes (Basel).* **2023**, 14(8), 1606.
18. Zhu, N.; Ren, H.; Yang, L.; Mao, G.; Li, J.; Su, C.; Yang, Y. Direct evidence of cheetah (*Acinonyx jubatus*) as intermediate host of *Toxoplasma gondii* through isolation of viable strains. *BMC Vet. Res.* **2024**, 20(1), 71.

Table S2. Genotypes of *Toxoplasma gondii* isolate from sheep by PCR-RFLP.

Isolated ID	SAG1	(3'+5') SAG2	Alt SAG2	SAG3	BTUB	GRA6	C22-8	C29-2	L358	PK1	Apico	ROP18	ROP5	Genotype ToxoDB
GT1	I	I	I	I	I	I	I	I	I	I	I	1	1	#10
PTG	II	II	II	II	II	II	II	II	II	II	II	2	2	#1
CTG	II / III	III	III	III	III	III	III	III	III	III	III	3	3	#2
TgCgCa1	I	II	II	III	II	II	II	u-1	I	u-2	I	2	5	#66
MAS	u-1	I	II	III	III	III	u-1	I	I	III	I	4	4	#17
TgCatBr5	I	III	III	III	III	III	I	I	I	u-1	I	4	4	#19
TgCatBr64	I	I	u-1	III	III	III	u-1	I	III	III	I	3	3	#111
TgRsCr1	u-1	I	II	III	I	III	u-2	I	I	III	I	3	3	#52
TgSheepCHn15	I	I	I	I	I	I	I	I	I	I	I	1	1	#10

Table S3. Isolation *Toxoplasma gondii* ToxoDB #10 (strains or DNA) from human and animals (1937-2024).

Country	*T. gondii* Strain (Year Isolated)	Source	Strains/DNA	Pathogenicity	References
USA	RH (1937)	Boy aged 6 years, B	Strain	Lethal for human, mice, guinea pigs, rhesus monkeys, rabbits, chicks	1
	GT1 (1978)	Adult female goat, Sk	Strain	Lethal for mice	2
	TgRshAL (1998)	Hawk, B	Strain	UK	3
Colombia	TgCtCo2, TgCtCo7 (2005)	Cat, B, H	Strains	TgCtCo7 were lethal for mice, TgCtCo2 was non-lethal for mice.	4,5
Mexico	TgCatMxQR1 (2012)	Cat, H, D	DNA	UK	6
Brazil	PS-TgCatBrSC1 (2015)	Five-year-old male cat, Lu	DNA	Lethal for cat	7
	TgCkBrSC1 (UK)	Free-range chicken, B, H	Strain	UK	8
Iran	TgAbIr16, TgAbIr18, TgAbIr19 (2014-2015)	Aborted fetus (sheep, goat, and cattle)	DNA	UK	9
China	TgCtxz1 (2011)	Cat, B, H	Strain	Lethal for mice	10
	TgHuZS2 (2011)	Human, Bl	Strain	UK	
	SH (UK)	Human, UK	Strain	UK	11
	CN, NT (UK)	Pig, UK	Strains	UK	
	KS (1983)	Human fetus	Strain	Lethal for mice	12
	Unnamed	Free-range chickens, B, H, Sp, Lu, Li, Ki	Strain	Lethal for mice	13
	HNP1 (2015-2017)	Pig, B	DNA	UK	14
	TgPHs1, TgpNx, TgPXx, TgPXd, TgPLh, Tgpxdb1, TgPxda, Tgpyh, TgPGZ, TgPNY, (2007-2009)	Sick pigs, hilar LN	DNA	UK	15
	P32, P75, P101 (2011-2016)	Sick pig, Li, Lu, H, LN, Ki	DNA	UK	16
	TgPJL1, TgPJL2, TgPJL3 (2013)	Slaughtered pig, hilar LN	DNA	UK	17
	FRP237 (2016)	Free-ranging pig, hilar LN	DNA	UK	18
	GZL56, GZL56 (UK)	Microtus Fortis, Lu	DNA	UK	19
	TgGYn1-TgGYn7 (2011-2014)	Black goat, Lu, Li, LN	DNA	UK	20
	Tg16 (2011)	Slaughtered cattle, Bl, Li	DNA	UK	21
	HNC39 (2019-2020)	Cattle, Sk from market	DNA	UK	22
	Tgdonkey226 (2015-2017)	Donkey, B	DNA	UK	23
	ZZC18, ZZC19, SMC20, SMC22, SMC30, SMC31, QZC68(UK)	Free-range chickens, Lu, Li	DNA	UK	24
	151, 153, 158 (2017)	Tree sparrows, B	DNA	UK	25
	S10 (UK)	Tree sparrows, breast muscle	DNA	UK	26
	Tgfox3, Tgfox4 (2014-2015)	Slaughtered Arctic fox, B	DNA	UK	27
	TgraccoonHB1 (2016-2017)	Slaughter Raccoon dog, B	DNA	UK	28
	TgBatCN1-2, TgBatCN 4-5 (2010-2011)	Bat, Li, In, Lu	DNA	UK	29
	TgBatJL3,TgBatGD9, TgBatGD10 (2005-2013)	Bat, Li	DNA	UK	30
	FZD58 (2021)	Market-sold duck, H	DNA	UK	31
	TgSheepCHn15 (2021)	Sheep, LN, Sp	Strain	Non-lethal for mice	Present study

UK: Unknown

B: brain; Bl: blood; D: diaphragm; H: heart; In: intestine; Li: liver; LN: lymph nodes; Lu: lung; Ki: kidney; Sp: spleen; Sk: skeletal muscle.

Table S3 References

1. Sabin, A.B. Toxoplasmic encephalitis in children. *J. Am. Med. Assoc.* **1941**, 116, 801-807.
2. Dubey, J.P. Mouse pathogenicity of *Toxoplasma gondii* isolated from a goat. *Am. J. Vet. Res.* **1980**, 41, 427-429.
3. Yu, L.; Shen, J.; Su, C.; Sundermann, C.A. Genetic characterization of *Toxoplasma gondii* in wildlife from Alabama, USA. *Parasitol. Res.* **2013**, 112(3), 1333-1336.
4. Dubey, J.P.; Su, C.; Cortés, J.A.; Sundar, N.; Gómez-Marín, J.E.; Polo, L.J.; Zambrano, L.; Mora, L. E.; Lora, F.; Jimenez, J.; Kwok, O. C.; Shen, S. K.; Zhang, X.; Nieto, A.; Thulliez, P. Prevalence of *Toxoplasma gondii* in cats from Colombia, South America and genetic characterization of *T. gondii* isolates. *Vet. Parasitol.* **2006**, 141: 42-47.
5. Rajendran, C.; Su, C.; Dubey, J.P. Molecular genotyping of *Toxoplasma gondii* from Central and South America revealed high diversity within and between populations. *Infect. Genet. Evol.* **2012**, 12(2), 359-368.
6. Valenzuela-Moreno, L.F.; Rico-Torres, C.P.; Cedillo-Peláez, C.; Luna-Pastén, H.; Méndez-Cruz, S.T.; Lara-Martínez, G.; Correa, D.; Caballero-Ortega, H. Mixed *Toxoplasma gondii* infection and new genotypes in feral cats of Quintana Roo, México. *Acta Trop.* **2019**, 193, 199-205.
7. Pena, H. F. J.; Evangelista, C. M.; Casagrande, R. A.; Biezus, G.; Wisser, C. S.; Ferian, P. E.; Moura, A. B.; Rolim, V. M.; Driemeier, D.; Oliveira, S.; Alves, B. F.; Gennari, S. M.,; Traverso, S. D. Fatal toxoplasmosis in an immunosuppressed domestic cat from Brazil caused by *Toxoplasma*

gondii clonal type I. *Rev. Bras. Parasitol. Vet.* **2017**, 26, 177-184.

8. Pena, H. F. J.; Alves, B. F.; Soares, H. S.; Oliveira, S.; Ferreira, M. N.; Bricarello, P. A.; Machado, T. M. P.; Castro, B. B. P.; Gennari, S. M. Free-range chickens from Santa Catarina state, southern Brazil, as asymptomatic intermediate hosts for *Toxoplasma gondii* clonal type I and typical Brazilian genotypes. *Vet. Parasitol. Reg. Stud. Reports.* **2018**, 13, 55-59.
9. Amouei, A.; Sarvi, S.; Mizani, A.; Hashemi-Soteh, M. B.; Salehi, S.; Javidnia, J.; Hosseini, S. A.; Amuei, F.; Alizadeh, A.; Shabanzade, S.; Gholami, S.; Daryani, A. Genetic characterization of *Toxoplasma gondii* in meat-producing animals in Iran. *Parasit Vectors.* **2022**, 15(1), 255.
10. Wang, L.; Chen, H.; Liu, D.; Huo, X.; Gao, J.; Song, X.; Xu, X.; Huang, K.; Liu, W.; Wang, Y.; Lu, F.; Lun, Z. R.; Luo, Q.; Wang, X.; Shen, J. Genotypes and mouse virulence of *Toxoplasma gondii* isolates from animals and humans in China. *PLoS One.* **2013**, 8(1), e53483.
11. Zhou, P.; Zhang, H.; Lin, R. Q.; Zhang, D. L.; Song, H. Q.; Su, C.; Zhu, X. Q. Genetic characterization of *Toxoplasma gondii* isolates from China. Parasitol. Int. **2009**, 58, 193-195.
12. Hou, Z.; Zhou, Y.; Liu, D.; Su, S.; Zhao, Z.; Xu, J.; Tao, J. Genotyping and virulence analysis of *Toxoplasma gondii* isolates from a dead human fetus and dead pigs in Jiangsu province, Eastern China. *Acta Parasitol*. **2018**, 63, 397-411.
13. Zhao, G.W.; Shen, B.; Xie, Q.; Xu, L.X.; Yan, R.F.; Song, X.K.; Adam H.I.; Li, X.R. Isolation and molecular characterization of *Toxoplasma gondii* from chickens in China. *J. Integr. Agric.* **2012**, 11, 1347-1353.
14. Gui, B. Z.; Zheng, W. B.; Zou, Y.; Lv, Q. Y.; Liu, M. T.; Li, F.; Yuan, A. W.; Li, R. C.; Liu, G. H. Molecular detection and genotyping of *Toxoplasma gondii* in pigs for human consumption in Hunan Province, China. *Foodborne Pathog. Dis.* **2018**, 15, 809-813.
15. Zhou, P.; Nie, H.; Zhang, L. X.; Wang, H. Y.; Yin, C. C.; Su, C.; Zhu, X. Q.; Zhao, J. L. Genetic characterization of *Toxoplasma gondii* isolates from pigs in China. *J. Parasitol.* **2010**, 96, 1027-1029.
16. Hou, Z. F.; Su, S. J.; Liu, D. D.; Wang, L. L.; Jia, C. L.; Zhao, Z. X.; Ma, Y. F.; Li, Q. Q.; Xu, J. J.; Tao, J. P. Prevalence, risk factors and genetic characterization of *Toxoplasma gondii* in sick pigs and stray cats in Jiangsu Province, eastern China. Infect. *Genet. Evol.* **2018**, 60, 17-25.
17. Jiang, H.H.; Wang, S.C.; Huang, S. Y.; Zhao, L.; Wang, Z. D.; Zhu, X. Q.; Liu, Q. Genetic characterization of *Toxoplasma gondii* isolates from pigs in Jilin Province, northeastern China. *Foodborne Pathog. Dis.* **2016**, 13, 88-92.
18. Zhang, X.X.; Jiang, R.L.; Zhang, N.Z.; Wang, C.R.; Tao, W.F.; Xu, P.; Ma, C.F.; Hou, G.; Ni, H.B. Molecular detection and genotyping of *Toxoplasma gondii* in free-ranging pigs in Northeastern China. *Infect. Genet. Evol.* **2018**, 63, 110-115.
19. Zhang, X.X.; Huang, S.Y.; Zhang, Y.G.; Zhang, Y.; Zhu, X.Q.; Liu, Q. First report of genotyping of *Toxoplasma gondii* in free-living *Microtus fortis* in northeastern China. *J. Parasitol.* **2014**, 100, 692–694.
20. Miao, Q.; Huang, S.Y.; Qin, S.Y.; Yu, X.; Yang, Y.; Yang, J.F.; Zhu, X.Q.; Zou, F.C. Genetic characterization of *Toxoplasma gondii* in Yunnan black goats (*Capra hircus*) in southwest China by PCR-RFLP. *Parasit Vectors.* **2015**, 8, 57.
21. Ge, W.; Sun, H.; Wang, Z.; Xu, P.; Wang, W.; Mu, G.; Wei, F.; Liu, Q. Prevalence and genotype of *Toxoplasma gondii* infection in cattle from Jilin Province, Northeastern China. *Vector Borne Zoonotic Dis.* **2014**, 14, 399-402.
22. Yi, X.L.; Yang, W.H.; Zheng, H.L.; Cao, M.L.; Xiong, J.; Chen, W.C.; Zhou, Y.J.; Li, F.; Zhu, X.Q.; Liu, G.H. Seroprevalence and molecular detection of *Toxoplasma gondii* and *Neospora caninum* in beef cattle and goats in Hunan province, China. *Parasit Vectors.* **2024**, 17(1), 195.
23. Zhang, X.X.; Shi, W.; Zhang, N.Z.; Shi, K.; Li, J.M.; Xu, P.; Zhao, Q.; Du, R. Prevalence and genetic characterization of *Toxoplasma gondii* in donkeys in northeastern China. *Infect. Genet. Evol.* **2017**, 54, 455-457.
24. Chu, M.J.; Huang, L.Y.; Miao, W.Y.; Song, Y.F.; Lin, Y.S.; Li, S.A.; Zhou, D. H. First molecular detection and genotype identification of *Toxoplasma gondii* in chickens from farmers' markets in Fujian Province, Southeastern China. *Pathogens*. **2023**,12(10), 1243.
25. Liu, M.T.; Jiang, W.X.; Gui, B.Z.; Jin, Y.C.; Yi, J.N.; Li, F., Zheng, W.B.; Liu, G.H. Molecular prevalence and genetic characterization of *Toxoplasma gondii* in wild birds in Hunan Province, China. *Vector Borne Zoonotic Dis*. **2019**, 19(5), 378-383.
26. Huang, S.Y.; Cong, W.; Zhou, P.; Zhou, D.H.; Wu, S.M.; Xu, M.J.; Zou, F.C.; Song, H.Q.; Zhu, X.Q. First report of genotyping of *Toxoplasma gondii* isolates from wild birds in China. *J. Parasitol.* **2012**, 98, 681-682.
27. Zhang, X.X.; Cong, W.; Ma, J.G.; Lou, Z.L.; Zhao, Q.; Meng, Q.F.; Qian, A.D.; Zhu, X.Q. First genetic characterization of *Toxoplasma gondii* infection in Arctic foxes (*Vulpes lagopus*) in China. *Infect. Genet. Evol.* **2016,** 44, 127-129.
28. Qin, S.Y.; Chu, D.; Sun, H.T.; Wang, D.; Xie, L.H.; Xu, Y.; Li, J.H.; Cui, D.Y.; You, F.; Cai, Y.; Jiang, J. Prevalence and genotyping of *Toxoplasma gondii* infection in raccoon dogs (*Nyctereutes procyonoides*) in northern China. *Vector Borne Zoonotic Dis.* **2020**, 20, 231-235.
29. Jiang, H.H.; Qin, S.Y.; Wang, W.; He, B.; Hu, T.S.; Wu, J.M.; Fan, Q.S.; Tu, C.C.; Liu, Q.; Zhu, X.Q. Prevalence and genetic characterization of *Toxoplasma gondii* infection in bats in southern China. *Vet. Parasitol.* **2014**, 203, 318-321.
30. Qin, S.Y.; Cong, W.; Liu, Y.; Li, N.; Wang, Z.D.; Zhang, F.K.; Huang, S.Y.; Zhu, X.Q.; Liu, Q. Molecular detection and genotypic characterization of *Toxoplasma gondii* infection in bats in four provinces of China. *Parasit Vectors.* **2014**, 7, 558.
31. Li, S.A., Huang, L.Y., Guo, X.D., Miao, W.Y., Lin, Y.S., Zhou, D.H. First identified *Toxoplasma gondii* Type I in market-sold Ducks in Fujian province, China: a significant for public health. Poultry Sci. **2024**, 103(9), 104024.

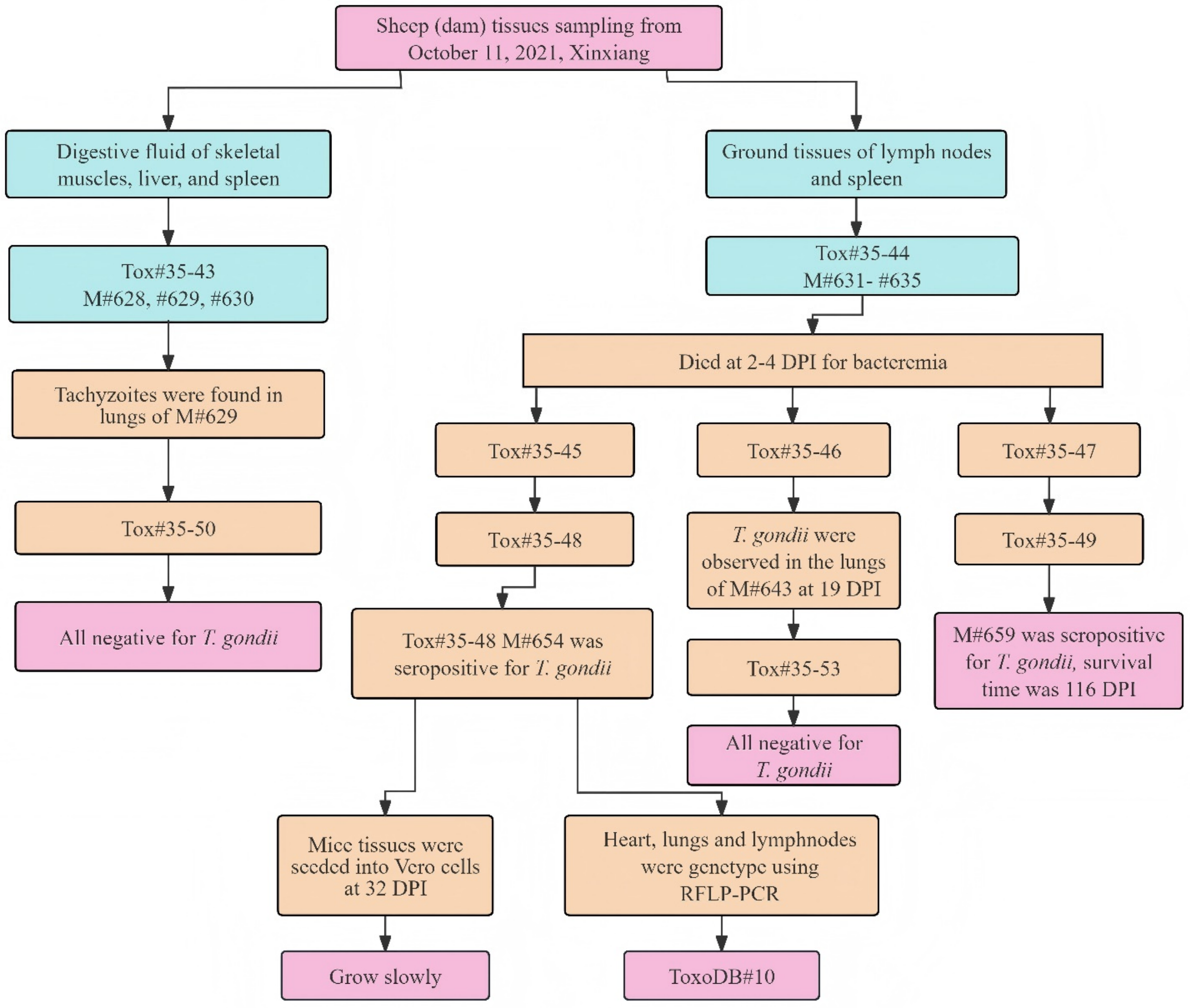

Figure S1. Flow chart for isolation of *Toxoplasma gondii* from sheep tissues.

DPI: days post-inoculation.

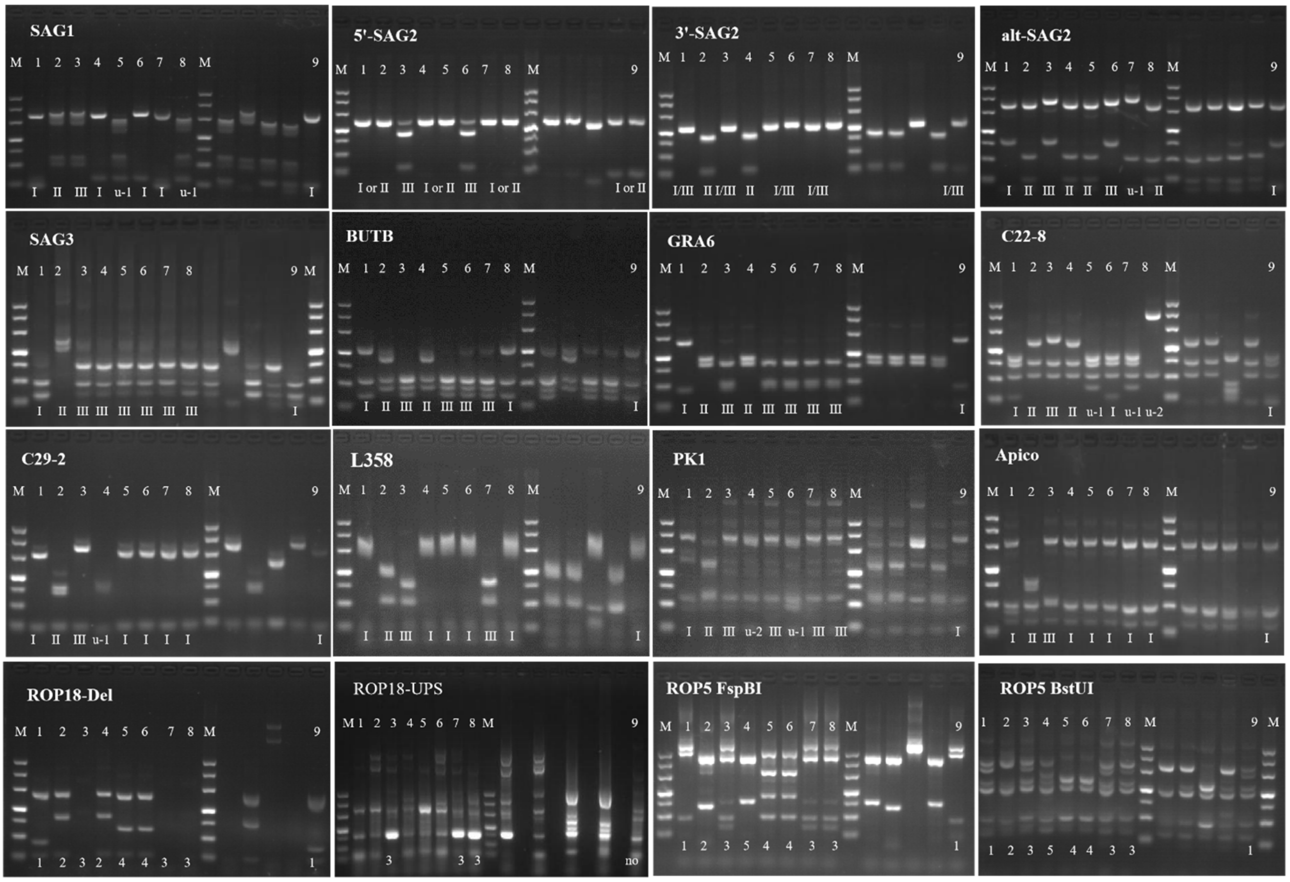

Figure S2. Genotypes of *Toxoplasma gondii* strain from sheep.

1: GT1, 2: PTG, 3: CTG, 4: TgCgCal, 5: MAS, 6: TgCatBr5, 7: TgCatBr64, 8: TgRsCr1,

9: TgSheepCHn15, M: Markers (500, 400, 300, **200**, 150, 100, 50).
